# Crucial Role of Perilipin-3 (TIP47) in Formation of Lipid Droplets and PGE_2_ Production in HL-60-Derived Neutrophils

**DOI:** 10.1371/journal.pone.0071542

**Published:** 2013-08-01

**Authors:** Fuyuki Nose, Tomohiro Yamaguchi, Rina Kato, Toshihiro Aiuchi, Takashi Obama, Shuntaro Hara, Matsuo Yamamoto, Hiroyuki Itabe

**Affiliations:** 1 Department of Periodontology, Showa University School of Dentistry, Tokyo, Japan; 2 Division of Biological Chemistry, Department of Molecular Biology, Showa University School of Pharmacy, Tokyo, Japan; 3 Division of Health Chemistry, Department of Healthcare and Regulatory Sciences, Showa University School of Pharmacy, Tokyo, Japan; The University of New South Wales, Australia

## Abstract

Cytosolic lipid droplets (LDs), which are now recognized as multifunctional organelles, accumulate in leukocytes under various inflammatory conditions. However, little is known about the characteristic features of LDs in neutrophils. In this study, we show that perilipin-3 (PLIN3; formerly called TIP47) is involved in LD formation and the inflammatory response in HL-60-derived neutrophils. HL-60, a promyelocytic cell line, was differentiated into neutrophils via treatment with all-*trans* retinoic acid. After differentiation, cells were stimulated with *Porphyromonas gingivalis* lipopolysaccharide (*P.g*-LPS), a major pathogen in adult periodontitis. When HL-60-derived neutrophils were stimulated with *P.g*-LPS, LDs increased in both number and size. In the differentiated cells, PLIN3 was induced while PLIN1, PLIN2 and PLIN5 were not detected. PGE_2_ production and the PLIN3 protein level were increased by the *P.g*-LPS treatment of the cells in a dose-dependent manner. When PLIN3 was down-regulated with siRNA treatment, LDs essentially disappeared and the level of PGE_2_ secreted in the cell culture medium decreased by 65%. In addition, the suppression of PLIN3 repressed the PGE_2_ producing enzymes; *i.e.*, microsomal PGE synthase-1, -2 and cyclooxygenase-2. These findings indicate that PLIN3 has a pivotal role in LD-biogenesis in HL-60-derived neutrophils, and that PLIN3 is associated with the synthesis and secretion of PGE_2_.

## Introduction

Cytosolic lipid droplets (LDs), also called lipid bodies or adiposomes, are functional organelles that are ubiquitously expressed in a variety of cells. LDs are composed of triacylglycerol (TG) and/or cholesterol esters, phospholipids and associated proteins present at the LD surface [1]–[4]. LDs are involved in multiple intracellular processes, including membrane biosynthesis, lipid metabolism and vesicle trafficking, and ultimately play an important role in the energy balance of the entire body [5], [6]. Although LDs are ubiquitously present in cells, the composition of the LD-associated proteins varies among cell types and changes under different physiological conditions. The LD-associated proteins contribute to the specific cellular functions of LDs. The perilipin family, conventionally called the PAT family, is a representative group of LD-associated proteins composed of five members. The recently proposed unified nomenclature designates them PLIN1 (the classic perilipin), PLIN2 (ADRP, ADFP, or adipophilin), PLIN3 (TIP47, PP17, or M6PRBP), PLIN4 (S3–12) and PLIN5 (MLDP, OXPAT, LSDP5, or PAT1) [7]. These proteins have two highly conserved regions; the PAT-1 domain locates at the N terminus and an 11-mer repeat locates at the C terminus and is named the PAT-2 domain [8], [9]. PLIN1 is the most abundant LD protein in adipocytes, and it acts as a hormone-dependent switch in TG hydrolysis [10]. PLIN2 is involved in the accumulation of LDs in various cell types [10], [11]. PLIN2 and PLIN3 are both expressed ubiquitously in the body, while PLIN2 is relatively highly expressed in hepatic cells. Little is presently known about the functions of PLIN3. PLIN4 is expressed in adipocytes, yet it does not locate on the large, PLIN1-positive LDs in untreated adipocytes. Instead, it is abundantly found on punctate structures in the cytoplasm [11]. PLIN5 is particularly abundant in the heart and is essential for maintaining LDs in the cardiac and skeletal muscle cells by antagonizing lipases [12]–[14].

PLIN3 was initially described as a tail-interacting protein of 47 kDa (TIP47) as well as a mannose-6-phosphate receptor-binding protein (M6PRBP). The trafficking of lysosomal hydrolases to prelysosomes is controlled by a modification with oligosaccharide chains and then the subsequent binding with the mannose-6-phosphate receptor (M6PR). TIP47 binds to the cytosolic domain of the transmembrane protein M6PR that is required for M6PR transport from the endosome to the *trans*-Golgi network, and thus TIP47 has been thought to have a role in the gathering of endosomal M6PR into transport vesicles for transfer back to the Golgi complex [15]. Recently, Bulankina *et al.* reported that TIP47 and M6PR located to different compartments in HeLa cells and the knockdown of TIP47 did not affect M6PR localization, questioning the effect of TIP47 function on lysosomal protein trafficking [16]. TIP47 was found to be one of the LD-associated proteins, since it shares sequence homology with the PAT family proteins, and proteomic analyses showed the presence of TIP47 in LDs [17]–[19], hence it was renamed PLIN3. Although PLIN3 shares the PAT domain with PLIN1 and PLIN2, PLIN3 is distributed in both LDs and the cytosol [5], and the functional reason for the difference in the distribution pattern of these proteins has not been elucidated. It has been proposed that PLIN3 has a hydrophobic cleft that is necessary for its association with LD lipids, explaining the accessibility of PLIN3 to LD [20], [21]. Alternatively, PLIN3 has been suggested to have a capacity to form disk-shaped lipid particles [16]. There obviously remains a great deal that is unknown about PLIN3, including roles related to lipid metabolism and/or cellular functions.

LDs in adipocytes, hepatic cells and macrophages have been studied extensively in relation to the metabolic control of lipids and metabolic diseases such as obesity and atherosclerosis. It has become evident that LDs are involved in various diseases and pathological conditions. For example, a severe skin malformation is caused by the loss of the LD-associated protein CGI-58 [22], hepatic LD is required for hepatitis C virus (HCV) infection [23] and the expression of PLIN1 and 2 increases in the liver of non-alcoholic steatohepatitis patients [24]. LDs are also exists in various types of inflammatory cells. In addition, it was shown that LDs in leukocytes, especially macrophages and eosionophils, increase during inflammation [25], [26], suggesting LDs may function as a major production site for prostaglandins (PG) and other lipid mediators [1], [25].

We used *Porphyromonas gingivalis* lipopolysaccharide (*P.g*-LPS) as the stimulating substance. *P. gingivalis* is a non-motile, Gram-negative, rod-shaped, anaerobic pathogenic bacterium. This bacterium is known as one of the major etiological causes of both the development and progression of periodontal diseases [27]. Inflammation following *P. gingivalis* infection leads to the destruction of periodontal tissues and the resorption of alveolar bone, and ultimately, tooth loss [28]. *P. gingivalis* has been reportedly found in atheromatous plaques [29], and infection with *P. gingivalis* in apoE-KO mice enhanced atherosclerotic lesion development [30], suggesting that oral infection is not just a local event, but possibly the cause of systemic diseases.

In the present work, we studied the LDs which formed in HL-60-derived neutrophils in response to an inflammatory stimulus. HL-60, a cell line derived from a patient with acute promyelocytic leukemia, can be differentiated into either neutrophils or monocytes by incubation with a variety of chemical inducers [31], [32]. Neutrophils are the most abundant white blood cells in mammals and form an essential part of the innate immune system. The presence of lipid bodies in neutrophils was reported more than 40 years ago. Initially they were described as inclusion bodies detected by staining with osmium tetroxide [33]. In 1989 fine microscopic observations together with biochemical studies showed them to be a type of LD [34]. However, little has been reported on the characteristics of LDs in neutrophils. We observed in the current study that PLIN3-associated LD was induced by LPS treatment of HL-60-derived neutrophils. Furthermore, siRNA experiments strongly suggest that PLIN3 has a significant role in LD biogenesis and PGE_2_ production.

## Materials and Methods

### Antibodies and Reagents

Rabbit polyclonal antibodies (pAb) against prostaglandin E synthases (PGES), mPGES-1, mPGES-2 and cPGES, were purchased from the Cayman Chemical Co. (Ann Arbor, MI, USA). Goat anti-PLIN3 pAb, goat anti-cyclooxygenase (COX)-1 pAb and rabbit anti COX-2 pAb were from Santa Cruz Biotechnology, Inc. (Santa Cruz, CA, USA). Guinea pig pAb against PLIN1, PLIN2 and PLIN5 were from Progen Biotechnik (Heidelberg, Germany). Alexa Fluor 488 donkey anti-goat IgG, Alexa Fluor 488 donkey anti-rabbit IgG and Alexa Fluor 568 donkey anti-goat IgG were from Invitrogen (San Diego, CA, USA). LPS from *P. gingivalis* and *E. coli* were purchased from Invivogen (San Diego, CA, USA). All-*trans* retinoic acid (AtRA) was purchased from Sigma (St. Louis, MO, USA).

### Cell Culture and Differentiation

The human promyelocytic leukemia cell line HL-60 was purchased from ATCC. Mouse Leydig tumor cell line MLTC1 and human hepatocellular carcinoma cell line Huh7 were purchased from RIKEN Health Research Resource Bank. HL-60 and MLTC1 were cultured in RPMI-1640 medium and Huh7 were cultured in DMEM:F-12 with 10% fetal bovine serum (FBS) (Gibco®, Life Technologies Co., Carlsbad, CA, USA) supplemented with 50 U/mL penicillin, 50 µg/mL streptomycin and 2 mM L-glutamine at 37°C in a humidified 5% CO_2_ atmosphere. HL-60 cells were induced to differentiate into mature neutrophils by incubation with 2 µM AtRA for four days and 82.9% of cells were differentiated into neutrophils judged by nitrobluetetrazolium reduction assay [31]. The amount of PGE_2_ in the culture medium was measured with a PGE_2_ EIA Kit (Cayman Chemical Co.) according to the manufacturer’s directions.

### Lipid Droplet Staining

The HL-60 cells were attached on a slide glass by cytospin centrifugation at 800 rpm for 3 min. The cells were fixed with 4% paraformaldehyde. LDs were then stained with BODIPY493/503 (Molecular probe®, Life Technologies Co., Carlsbad, CA, USA). The number and area of the BODIPY-labeled LDs were calculated using ImageJ software (NIH, Bethesda, MD, USA).

### Lipid Analysis

Total lipids were extracted from HL-60 cells with chloroform/methanol/phosphate buffered saline (PBS) (1∶1:0.9) and then dissolved in chloroform. The lipids in the cells were separated on thin-layer chromatography (TLC) developed with petroleum ether/diethyl ether/acetic acid (90∶9:1, v/v/v) and then visualized with iodine vapor. The band intensity was calculated using ImageJ software (NIH, Bethesda, MA, USA).

### Immunofluorescence Analysis

After being treated with or without *P.g*-LPS (10 µg/mL) for 12 h, HL-60 cells were adhered to the microscope slide by cytospin and were fixed with 4% paraformaldehyde for 10 min. The cells were then permeabilized with 0.01% digitonin in PBS for 20 min and blocked with 2% FBS in PBS for 1 h. Cells were incubated with a primary pAb against PLIN3, mPGES-1, mPGES-2, cPGES or COX-2 for 1 h at 37°C. After washing with PBS, cells were then incubated with Alexa Fluor 488- or 568- conjugated secondary antibodies for 1 h at 37°C. After a PBS wash, the cells were stained with Hoechst33258 for 10 min to visualize the nuclei, then the cells were observed under confocal microscopy (A1-si; Nikon).

### Subcellular Fractionation

MLTC1 and Huh7 cells harvested from five 100 mm dishes were washed with PBS, resuspended in a hypotonic medium (20 mM Tris-Cl (pH 7.4), 1 mM EDTA, 10 mM sodium fluoride, with protease inhibitor cocktail) and incubated for 10 min on ice. Cells were homogenized by nitrogen cavitation. The homogenate was centrifuged at 1,000 ×g for 5 min at 4°C to obtain post-nuclear supernatant (PNS). For density gradient ultracentrifugation, PNS was adjusted to 20% sucrose and on top of that were layered buffers containing 5% sucrose and without sucrose. Centrifugation was carried out at 14,500 ×g for 1 h at 4°C. LD fractions were collected from the top and delipidated with cold acetone overnight at −20°C.

### Western Blot Analysis

Whole cell lysates derived from HL-60 neutrophils and the LDs of MLTC1 and Huh7 were subjected to 10% or 15% SDS-PAGE, and then transferred to polyvinylidene difluoride membranes. After blocking with 5% skim milk in PBS containing 0.1% Tween-20 (PBST), the membranes were incubated with a primary pAb in 1% skim milk overnight at 4°C. After washing with PBST, membranes were incubated with horseradish peroxidase-conjugated secondary pAb for 2 h at room temperature. The bands were detected with ECL-plus Western blotting detection reagent (GE Healthcare UK Ltd., Buckinghamshire, UK) and then visualized using X-ray film (RX-U; Fuji Film Co., Tokyo, Japan).

### Transfection with Small Interfering RNA (siRNA)

Synthetic siRNA probes were purchased from Thermo Scientific (Chicago, USA). After treatment with 2 µM AtRA for four days, HL-60 cells (1×10^7^ cells) were transfected with siRNA for human M6PRBP1 and non-targeting siRNA (ON-TARGETplus SMARTpool L-015979-00-0005 and #1 D-001810-01-05, respectively) at a final concentration of 250 nM using a Nucleofector™ electroporator (Amaxa biosystems, Germany) with Nucleofector Kit V (Lonza, Germany) according to the manufacturer’s directions. After transfection with siRNA, cells were cultured for 72 h and then cells were cultured in a new medium containing with *P.g*-LPS (10 µg/mL) for 12 h.

### Statistical Analysis

Data are expressed as the mean ± standard deviation. Results were analyzed using Student’s *t*-test and Dunnet’s test. Statistical significance for all comparisons was assigned at P<0.05. Asterisks indicate values significantly different from the control: *P<0.05.

## Results

### LD Formation and TG Accumulation in HL-60-derived Neutrophils are Induced by *P.g*-LPS

**Figure 1:**
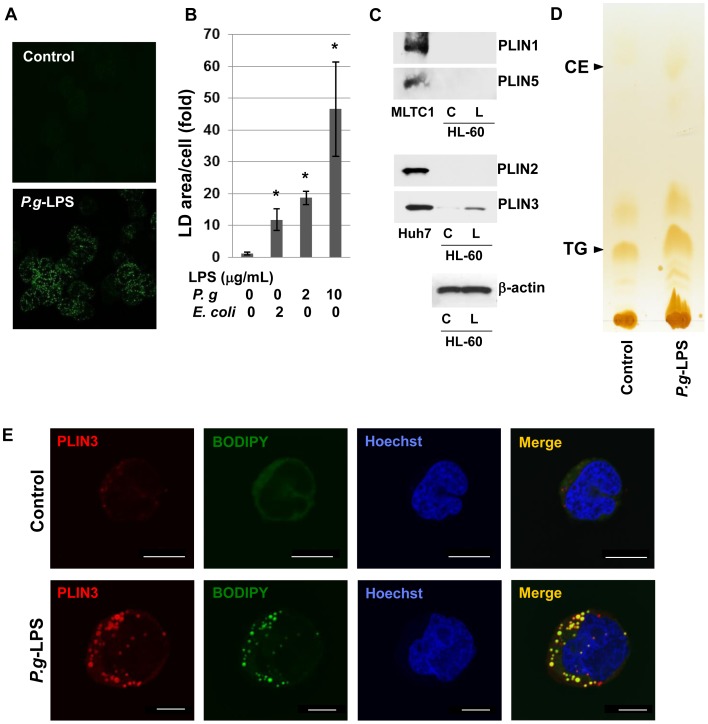
The size and number of LDs in HL-60-derived neutrophils were increased by *P.g*-LPS. A: HL-60 cells were differentiated into neutrophils by treatment with 2 µM AtRA for 4 days and then cultured with or without 10 µg/mL *P.g*-LPS for 12 h. Cytosolic LDs were labeled with BODIPY493/503 and observed using confocal laser microscopy. B: BODIPY-stained LDs were quantified by the area of fluorescence per cell using NIH ImageJ software. The mean ± S.E. from at least three images were calculated. C: HL-60 neutrophils were incubated in the absence (C) or presence (L) of 10 µg/mL *P.g*-LPS for 12 h. The expression levels of PLIN1, PLIN2, PLIN3 and PLIN5 were detected by Western blotting. LDs recovered from the MLTC1 cells were used as a positive control for PLIN1 and PLIN5, and Huh7 LDs were for PLIN2 and PLIN3. D: HL-60 neutrophils were incubated in the absence or presence of 10 µg/mL *P.g*-LPS for 12 h. Total lipids extracted from the cells were separated on thin-layer chromatography. The lipids were visualized with iodine vapor, and the band intensity of TG was calculated using ImageJ. E: Differentiated HL-60 cells were treated with or without 10 µg/mL *P.g*-LPS for 12 h. Cells were fixed and labeled with BODIPY493/503 (green) and anti-PLIN3 pAb (red). Nuclei were stained with Hoechst33258. The cells were observed under confocal laser microscopy.

HL-60 cells were treated with AtRA to inhibit the clonal growth and induce differentiation into neutrophils. After four days of incubation with 2 µM AtRA, HL-60-derived neutrophils were incubated with *P.g*-LPS for 12 h, then LD was visualized by staining the cells with BODIPY493/503. The number of LDs and LD area were analyzed using ImageJ software. There were almost no LDs observed in HL-60-derived neutrophils without any stimulation, but a number of LDs appeared after *P.g*-LPS treatment (Fig. 1A). The total area of LD after the stimulation with 10 µg/mL *P.g*-LPS resulted in a 38-fold increase compared with the LD in the non-stimulated cells (Fig. 1B). The effect of *P.g*-LPS on LD formation was stronger than LPS from *E. coli*. Since 10 µg/mL *E. coli*-LPS caused cytotoxicity, we used *P.g*-LPS throughout this study. TLC analysis of the total lipid extract in the HL-60-derived neutrophils revealed that the major lipid which accumulated in the stimulated cells was TG and that TG increased 2.8-fold by stimulation with *P.g*-LPS (Fig. 1D).

The PLIN family proteins coat the surface of intracellular LDs in various cell types. Western blot analyses demonstrated that the *P.g*-LPS treatment of HL-60-derived neutrophils induced PLN3, but other well-known PLIN family proteins, PLIN1, PLIN2 and PLIN5, were not detected (Fig. 1C). MLTC1 cells and Huh7 cells were used as a positive control for perilipin family proteins. It was observed microscopically that PLIN3 colocalized with BODIPY-stained LDs in the *P.g*-LPS treated cells (Fig. 1E). After 12 h stimulation of HL-60-derived neutrophils, *PLIN3* mRNA did not increase (0.82±0.31 fold of non-stimulated cells), although the data has a large error. These results suggest that PLIN3-associated LDs were induced in the stimulated HL-60 neutrophils.

### The Expression of PLIN3 and PGE_2_ Production was Induced by *P.g*-LPS

**Figure 2:**
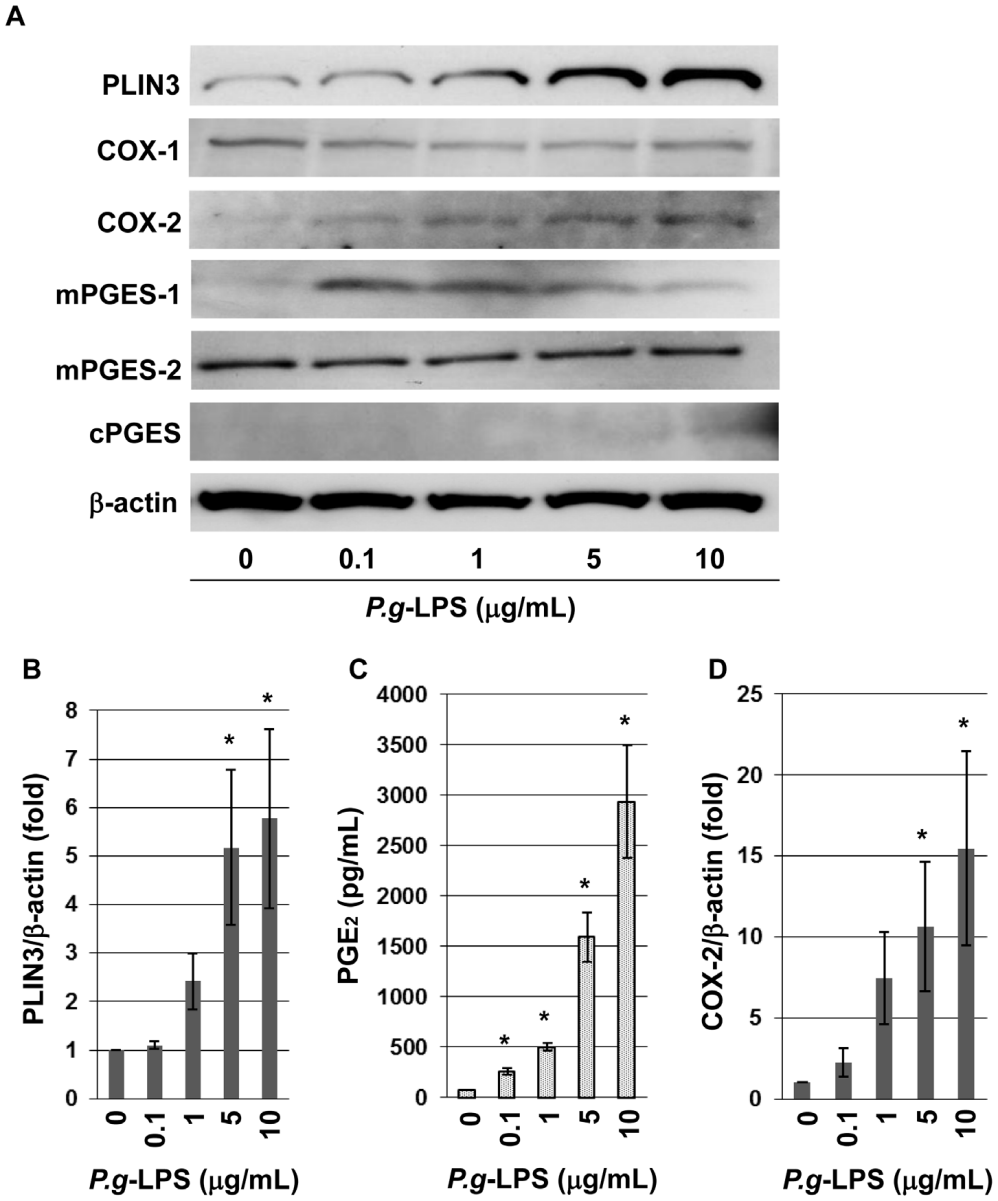
PLIN3 and PGE_2_ production were increased by *P.g*-LPS treatment in a dose-dependent manner. HL-60-derived neutrophils were incubated with or without *P.g*-LPS for 12 h at the concentrations indicated. A: Expression levels of PLIN3, mPGES-1, mPGES-2, COX-1, COX-2, β-actin in HL-60-neutrophils were detected by Western blotting. B: The level of PLIN3 expression was determined using ImageJ software. C: The levels of PGE_2_ into the media were measured by EIA. D: The level of COX-2 expression was determined using ImageJ. Data are the mean ± SD of three independent experiments. * P<0.05 (Dunnett’s test, vs control).

To investigate the inflammatory responses in the cells, the amount of PGE_2_ in the culture medium was measured by enzyme-linked immunoassay. HL-60-derived neutrophils were treated with various concentrations of *P.g*-LPS for 12 h. Western blot analysis revealed that the expression of PLIN3 was increased by *P.g*-LPS in a concentration-dependent manner (Fig. 2A, B). It was clearly shown that *P.g*-LPS treatment induced PGE_2_ production, and the amount of PGE_2_ increased in accord with the *P.g*-LPS concentration (Fig. 2C).

PGE_2_ is produced from arachidonic acid by cyclooxygenase (COX) and PGE_2_ synthase (PGES), with each of these enzymes having several isozymes. We examined the changes in the expression of mPGES-1, mPGES-2, COX-1 and COX-2 by Western blot analysis, and found that only COX-2 was increased concomitantly with PLIN3 induction by *P.g*-LPS in a concentration-dependent manner (Fig. 2A, D). The enzyme mPGES-1 was induced by *P.g*-LPS, but was suppressed at the highest concentration.

**Figure 3:**
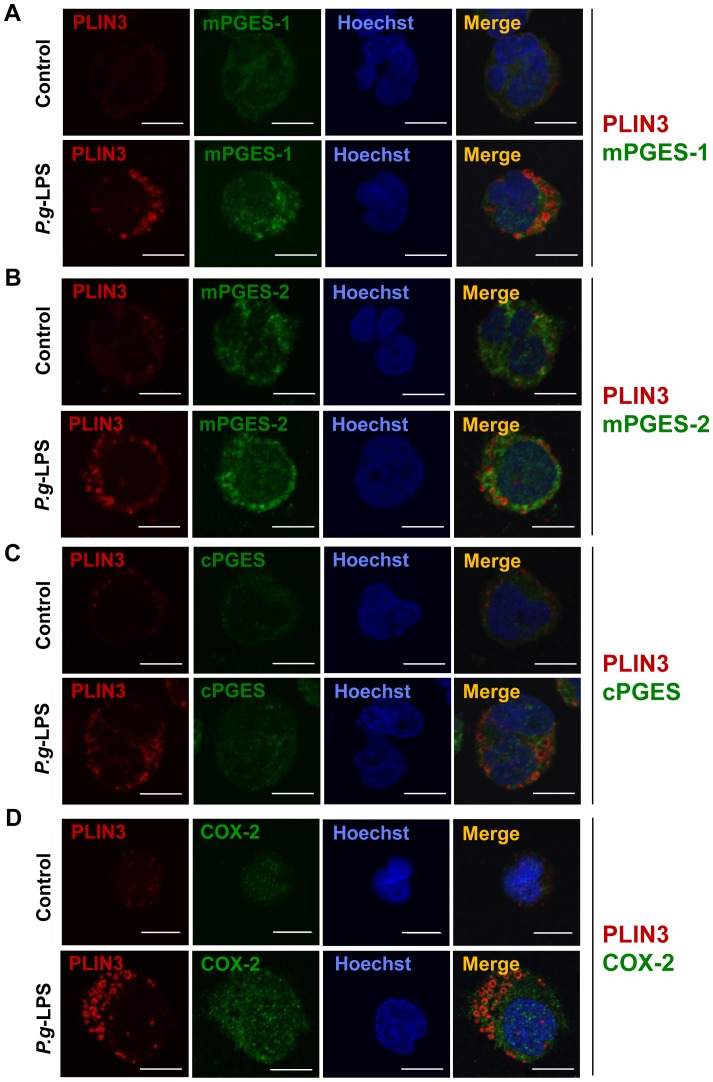
PLIN3 and PGE_2_ synthesizing enzymes induced in the *P.g*-LPS-treated HL-60 neutrophils distribute differently. Differentiated HL-60 cells were treated with or without 10 µg/mL *P.g*-LPS for 12 h. Cells were fixed and then double stained with anti-PLIN3 and either anti-mPGES-1, anti-mPGES-2, anti-cPGES or COX-2 pAbs. Nuclei were stained with Hoechst33258. The cells were observed under confocal laser microscopy.

Differentiated HL-60 cells treated with or without *P.g*-LPS were immunostained and observed microscopically. *P.g*-LPS treatment increased the expression of PLIN3 under the same conditions that the expressions of mPGES-1 and COX-2 were induced, but mPGES-2 and cPGES were largely unaffected (Fig. 3). These observations correspond well with those in Fig. 2. The intracellular distribution of PLIN3 did not match that of the PGE_2_ synthesizing enzymes.

### The Knockdown of PLIN3 Reduces LD Formation and the Production of PGE_2_

**Figure 4:**
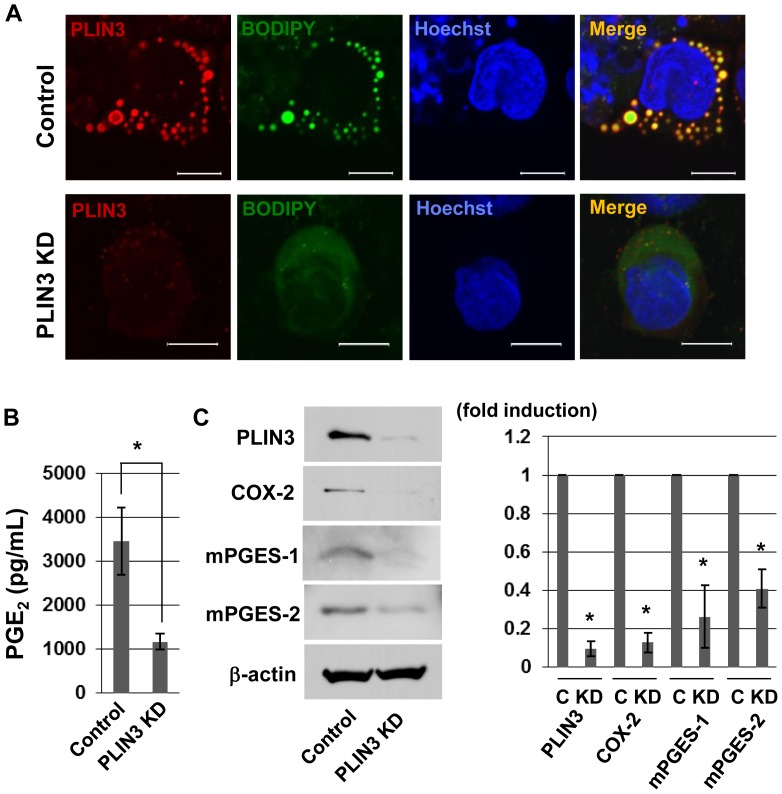
PLIN3 knockdown in HL-60-derived neutrophils suppressed the formation of LDs and production of PGE_2_. A: After treatment of HL-60-derived neutrophils with PLIN3 siRNA or control siRNA for 72 h, the cells were stimulated with 10 µg/mL *P.g-*LPS for 12 h. The cells were fixed and labeled with BODIPY493/503 and anti-PLIN3 pAb. Bar = 10 mm. B: Cells were treated as described for A, and the PGE_2_ released into the media was measured by EIA. C: Whole cell lysates were subjected to SDS-PAGE and the protein levels of mPGES-1, mPGES-2, COX-2 and β-actin were analyzed by Western blotting. The band intensities were calculated by ImageJ software. Data are the mean ± SD of three independent experiments. *P<0.05.

To determine whether PLIN3 is related to the production of PGE_2_, RNAi experiments were carried out to knock down PLIN3 expression. Cells transfected with either PLIN3 siRNA or control siRNA were stimulated with AtRA. LDs essentially disappeared in the cells when treated with PLIN3 siRNA (Fig. 4A). PGE_2_ production was significantly decreased by PLIN3 knockdown (Fig. 4B). Furthermore, the suppression of PLIN3 reduced the protein levels of mPGES-1, mPGES-2 and COX-2 by 75%, 59% and 90%, respectively compared with non-targeting siRNA (Fig. 4C).

**Figure 5:**
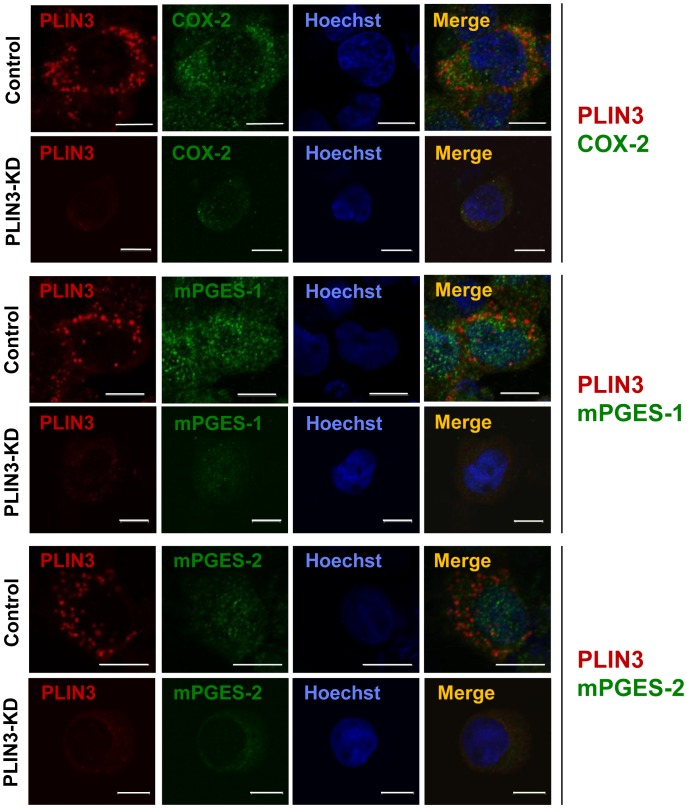
PLIN3 knockdown reduced the PGE_2_ synthesizing enzymes. After PLIN3 or control siRNA treatment of HL-60-derived neutrophils for 2 days, cells were stimulated with *P.g-*LPS for 12 h, as in Fig. 4. Cells were fixed and then double stained with anti-PLIN3 and either COX-2, anti-mPGES-1, or anti-mPGES-2 pAbs. Nuclei were stained with Hoechst33258. The cells were observed under confocal laser microscopy.

We next investigated the behavior of these PGE_2_ producing enzymes, when PLIN3 expression was knocked down. Fig. 5 shows that the suppression of PLIN3 leads to the reduction of mPGES-1, mPGES-2 and COX-2, which is in good correspondence with the results of Western blot (Fig. 4).

## Discussion

Recently LDs have been paid attention as a functional organelle related to cellular lipid metabolism. LD-associated proteins have been extensively studied and it is well known that the PAT family proteins are the major LD-associated proteins in a variety of cell types, however, little is presently known about the functions of PLIN3. To the best of our knowledge this is the first to report PLIN3 is a crucial protein component of LD involved in LD formation and PGE_2_ production in HL-60-derived neutrophils.

Some of the LD-associated proteins have been extensively studied. In particular, PLIN1 is a primary LD-associated protein in adipocytes, and it has a crucial role in the regulation of lipolysis by associating with CGI-58 and adipose TG lipase [23], [35]. PLIN2, which is highly expressed in hepatocytes, macrophages and premature adipogenic fibroblasts, is considered to be expressed ubiquitously. The expression level of PLIN2 is closely related to lipid accumulation in cells, indicating it has a role in the formation of the LD structure [36], [37]. PLIN5 is specifically expressed in cardiac and skeletal muscles as major LD-associated protein [12]. LDs in muscle cells are generally small and located in very proximity with mitochondria. PLIN5-KO mice exhibit intolerance to energy expenditure, and PLIN5 plays a crucial role as a distributor of the energy for β-oxidation [38]. Although PLIN3 and PLIN4 are members of the PAT family proteins, their functions have yet to be clarified.

PLIN3 and PLIN2 are expressed abundantly in macrophages and other types of cells, and their expression is thought to be ubiquitous [5], [39]. It is a surprising observation in this study that PLIN3 was found to be the predominant PAT protein in HL-60-derived neutrophils while PLIN2 was under the detection limit. In addition, *P.g*-LPS treatment induced LD formation as well as PLIN3 expression, and the suppression of PLIN3 by siRNA dramatically reduced LD formation. These results strongly suggest that PLIN3 has an ability to both generate and maintain LDs. In our preliminary experiments, both PLIN2 and PIN3 were expressed in human neutrophils from peripheral blood, suggesting HL-60 may be lacking in PLIN2.

Buers, *et al.* reported that LD-associated PLIN3 increased when PLIN2 was suppressed by siRNA treatment in THP-1 macrophages [40]. This observation can be explained if PLIN3 has a capacity to compensate for the loss of PLIN2 in maintaining the LD structure. The absence of PLIN2, which could compensate the role of PLIN3, is likely to be one reason that PLIN3 is crucial for the formation of LD in HL-60-derived neutrophils. In THP-1 macrophages knockdown of PLIN3 reduced the cellular amount of TG by 10–20%, but had no effect on cholesterol [40], suggesting that PLIN3 plays only a limited role in lipid accumulation in macrophage LDs. Interestingly, the intracellular distribution of PLIN2 and PLIN3 overlapped only slightly, and knockdown of PLIN2 reduced cholesterol rather than TG [41]. A very recent report showed that PLIN3 was induced by treatment of RAW264.7 macrophages with insulin but not with free fatty acids [42]. The LD particles formed with PLIN3 appear to be regulated differently from PLIN2-dependent LD.

While *E. coli*-LPS is recognized by toll-like receptor 4 (TLR4), *P.g*-LPS is reported to activate TLR2 [43]. Recently, Gu, *et al.* reported that PLIN3 expression was induced by a TLR-9 stimulating compound, ODN1826, in RAW264.7 murine macrophages [41]. The stimulating pathways for PLIN3 expression may be different in different cell types.

Another finding of this study is that suppression of the PLIN3 protein dramatically reduced PGE_2_ production. In the literature, PGE_2_ production, which is carried out through three enzymatic steps, is thought to take place in intracellular membranes including the endoplasmic reticulum and perinuclear membranes [44]. Recent reports have suggested that PGE_2_ might be produced in LDs from the observation that some of the PGE_2_ producing enzymes co-localize with the PAT family proteins in macrophages or eosinophils [25], [45]. In this study we examined the co-localization of PGE_2_ producing enzymes with PLIN3 carefully, however, we did not observe any evidence that PLIN3 co-localizes with PGE_2_ synthesizing enzymes.

COX-2 is known to be an inducible protein and is a rate-limiting enzyme in PGE_2_ production. Under our experimental conditions, COX-2 was up-regulated by the *P.g*-LPS stimulus, and strongly suppressed by knockdown of PLIN3 expression. The mechanism of COX-2 regulation by PLIN3 is not clear yet. One possibility is that LD may be needed for the organization of the membrane domains on ER and/or nuclear membranes that in turn stabilizes the PGE_2_ producing enzymes. This would explain the result that not only COX-2, but also the mPGES-1 and mPGES-2 proteins were all suppressed by knockdown of PLIN3. Alternatively, since PLIN3 is actually involved in the sorting of lysosomal proteins [15], [16], it may control the trafficking of some proteins between intracellular membranes and LDs.

In conclusion, this study provides evidence for a functional role of PLIN3 in LD formation and PGE_2_ production. Further study is needed to answer a number of unresolved issues. We would like to obtain insight into the mechanisms by which PLIN3 regulates LD biogenesis and expression of PGE_2_-producing enzymes by identifying proteins which interact with PLIN3, for example, using proteomic analysis.
